# Children’s Sleeping

**Published:** 1872-06

**Authors:** 


### CHILDREN'S SLEEPING.

SOMETIMES children, who have gone to bed apparently well, wake up after a while in the greatest terror, making exclamations as if some fearful thing was impending; so alarmed sometimes that the parents are not recognized, and distressing shrieks alarm the whole house, sometimes subsiding in a few minutes into a sound sleep. This affection is almost always the result of costiveness, of not having gone to the privy for several days, or from derangement of the stomach in consequence of eating too heartily, or of a late supper. The best treatment is to soothe the child, take it into the lap, and rock it awhile, or change the position of the body in the bed so as to start the circulation. Wipe the face and hands with a soft rag dipped in cold water, and let the child have its sleep out in the morning. These attacks may come on every night or two for weeks and months, but can be easily prevented by promptly using some of the means ordinarily employed to secure a daily action of the bowels; by eating thrice a day, nothing between meals, and only bread and butter with weak tea for supper, and spending most of the day out of doors in active play and amusements.

